# Role of lncRNA MAGI2‐AS3 in lipopolysaccharide‐induced nucleus pulposus cells injury by regulating miR‐374b‐5p/interleukin‐10 axis

**DOI:** 10.1002/iid3.772

**Published:** 2023-04-17

**Authors:** Jiang Yu, Chengjin Li

**Affiliations:** ^1^ Department of Orthopedics Surgery Affiliated Hospital of Jianghan University Wuhan China

**Keywords:** extracellular matrix, intervertebral disc degeneration, nucleus pulposus cells

## Abstract

**Background:**

Intervertebral disc degeneration (IDD) is a pathological process that occurs during the natural aging of intervertebral discs. Accumulating evidence suggests that noncoding RNAs (ncRNAs), including microRNAs and long ncRNAs (lncRNAs), participate in the pathogenesis and development of IDD. Herein, we examined the role of lncRNA MAGI2‐AS3 in the pathogenic mechanism of IDD.

**Material and Methods:**

To develop an IDD in vitro model, we treated human nucleus pulposus (NP) cells with lipopolysaccharide (LPS). Aberrant levels of lncRNA MAGI2‐AS3, miR‐374b‐5p, interleukin (IL)‐10 and extracellular matrix (ECM)‐related proteins in NP cells were examined using reverse transcription‐quantitative PCR and western blot analysis. LPS‐induced NP cell injury and inflammatory response were confirmed using the MTT assay, flow cytometry, Caspase3 activity, and enzyme‐linked immunosorbent assay. Dual‐luciferase reporter assay and rescue experiments were performed to confirm targets between lncRNA MAGI2‐AS3 and miR‐374b‐5p or miR‐374b‐5p and IL‐10.

**Results:**

LPS‐induced NP cells exhibited low levels of lncRNA MAGI2‐AS3 and IL‐10 expression, along with high miR‐374b‐5p expression. miR‐374b‐5p was a target of lncRNA MAGI2‐AS3 and IL‐10. LncRNA MAGI2‐AS3 ameliorated injury, inflammatory response, and ECM degradation in LPS‐treated NP cells by downregulating miR‐374b‐5p to upregulate IL‐10 expression.

**Conclusions:**

LncRNA MAGI2‐AS3 increased IL‐10 expression levels by sponging miR‐374b‐5p, which, in turn, alleviated LPS‐triggered decreased NP cell proliferation and increased apoptosis, inflammatory response, and ECM degradation. Therefore, lncRNA MAGI2‐AS3 may be a potential therapeutic target for IDD.

## Graphical abstract

The present study aimed to expound the role of lncRNA MAGI2‐AS3 in the pathogenic mechanism of IDD. Our findings revealed that LncRNA MAGI2‐AS3 increased IL‐10 level by sponging miR‐374b‐5p, which in turn alleviated LPS‐triggered decrease in NP cell proliferation, increase in apoptosis, inflammatory response and extracellular matrix degradation. Therefore, lncRNA MAGI2‐AS3 may be a potential therapeutic target for IDD.

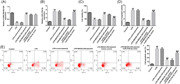

## 1 INTRODUCTION

Intervertebral disc degeneration (IDD) is an important pathological basis in the development of degenerative spinal diseases, such as lumbar spinal stenosis, lumbar disc herniation, and cervical spondylosis.^1^ The regulatory mechanisms underlying IDD are complex and modulated by multiple factors, including biomechanical, immune‐mediated damage, and nutritional factors.^2^ Furthermore, aging, trauma, and overweight are considered risk factors contributing to IDD.^3^, ^4^ These factors eventually induce the secretion of mediators such as tumor necrosis factor (TNF)‐α, interleukin (IL)‐1β, and nitric oxide (NO), resulting in increased extracellular matrix (ECM) degradation.^1^, ^5^ Furthermore, this disruption of the dynamic balance between ECM synthesis and degradation contributes to decreased type II collagen (collagen II) in the nucleus pulposus (NP) cells, reducing the water content and proteoglycans (PGs) in the disc, ultimately leading to IDD.^6^, ^7^ However, the specific molecular mechanisms underlying IDD need to be further explored.

Long noncoding RNAs (lncRNAs) are a class of RNA molecules more than 200 nt units in length that can regulate gene expression at post‐transcriptional, epigenetic, and transcriptional levels.^8^ In recent years, the impact of abnormal lncRNA expression on disease development has received growing attention. Emerging evidence has shown that epigenetic mechanisms, including lncRNAs, play an important role during osteoblastogenesis and osteoclastogenesis processes.^9^ Looking into the future, lncRNA can be considered as a potential innovative molecular biomarker, which is helpful for early diagnosis of bone metabolism related diseases and development of new therapeutic strategies. With the rapid development of sequencing and microarray technologies, several lncRNAs with substantial differential expression in IDD have been identified.^10^, ^11^, ^12^ Based on these findings, it can be suggested that lncRNAs are differentially expressed in the degenerated NP tissues and may participate in modulating the pathogenesis of IDD. In addition, research shows that lncRNAs can provide candidate diagnostic biomarkers as well as novel therapeutic strategies for IDD.^13^, ^14^ A rencent study indicated that lncRNA MAGI2‐AS3 is downregulated in IDD patients and participates in the modulation of FasL expression in NP cells.^15^ However, the mechanism related to the regulation of IDD progression by lncRNA MAGI2‐AS3 has not been examined and needs to be ascertained using in‐depth experiments.

LncRNAs can inhibit the expression of microRNAs (miRNAs) by functioning as endogenous miRNA sponges, which, in turn, alleviates the repression of target genes, enhancing their expression levels.^16^ miRNAs are a category of endogenous noncoding small molecule RNAs, averaging 22 nt in length.^17^, ^18^ Growing evidence suggests that miRNAs play a role in regulating IDD development, such as NP cell senescence, apoptosis, matrix and collagen degradation.^19^, ^20^, ^21^ miR‐374b‐5p has been studied in osteoporosis, and its antagonism can promote bone formation.^22^ Moreover, through bioinformatics analysis, we found that there was a binding site between miR‐374b‐5p and lncRNA MAGI2‐AS3. However, the role of miR‐374b‐5p in IDD development, and whether lncRNA MAGI2‐AS3 can regulate miR‐374b‐5p levels to modulate IDD progression warrants comprehensive evaluation.

More and more evidence shows that during the progress of IDD, NP cells are excessively apoptotic and ECM is excessively degraded.^23^, ^24^ In addition, inflammation, especially the abnormal excretion of inflammatory cytokines (TNF‐α, IL‐1β, and IL‐6) contributes to the occurrence and progress of IDD.^25^ Lipopolysaccharide (LPS) can induce apoptosis, inflammation and ECM degradation of NP cells, so the NP cells treated with LPS have been widely used in the study of IDD in vitro.^26^

In the present study, we aimed to evaluate the role and molecular mechanism of lncRNA MAGI2‐AS3 in an in vitro model of IDD by exposing NP cells to LPS.

## 2 MATERIALS AND METHODS

### 2.1 Cell culture, LPS treatment, and cell transfection

Human NP cells were purchased from American Type Culture Collection (ATCC) and cultured in Dulbecco's Modified Eagle Medium (DMEM)/F12 medium (with 10% fetal bovine serum and 1% penicillin‐streptomycin) at 37°C using a 5% CO_2_ incubator. Cells were cultured in monolayer and the 2−3 generations of cells were used in the following experiments. Experiments were performed for three times.

To construct an in vitro model of IDD, we treated NP cells with 10 ng/ml of LPS for 24 h.

The lncRNA MAGI2‐AS3 sequence was synthesized based on the lncRNA MAGI2‐AS3 sequence and then subcloned into the pcDNA3.1 vector (MAGI2‐AS3‐plasmid; Shanghai GeneChem Co., Ltd.). The empty pcDNA3.1 vector was used as a control (control‐plasmid). For IL‐10 knockdown, the control‐siRNA (sc‐36869) and IL‐10‐siRNA (sc‐39634) were purchased from Santa Cruz Biotechnology. NP cells were transfected with control‐plasmid, MAGI2‐AS3‐plasmid, control‐siRNA, IL‐10‐siRNA, mimic control (Shanghai GenePharma), miR‐374‐5p mimic (Shanghai GenePharma), inhibitor control (Shanghai GenePharma), miR‐374‐5p inhibitor (Shanghai GenePharma), MAGI2‐AS3‐plasmid+mimic control, MAGI2‐AS3‐plasmid + miR‐374‐5p mimic, miR‐374‐5p inhibitor+control‐siRNA, or miR‐374‐5p inhibitor + IL‐10‐siRNA using Lipofectamine 2000 reagent (Thermo Fisher Scientific, Inc.) at 37°C for 24 h as per the manufacturer's protocol.

### 2.2 RT‐qPCR assay

After cell transfection, total RNA was extracted from NP cells using the RNAiso Plus (9108Q; Takara), according to the manufacturer's instructions. Then, cDNA was obtained using the Takara RNA PCR Reverse Transcription Kit (6110A, Takara) after RNA quantification with NanoDrop2000. Next, cDNA was used as a template for PCR reactions using the TB Green® Fast qPCR Mix (RR430S, TaKaRa) with an ABI 7500 Real‐Time PCR System (Applied Biosystem) according to the manufacturer's protocol. The amplification conditions were as following: denaturation at 95°C for 10 min, followed by 40 cycles (95°C for 10 s, 60°C for 20 s, and 72°C for 30 s). GAPDH or U6 served as internal references. Finally, the experimental data were normalized and quantified by the $2^{‐△△C_{T}}$ method. Primer sequences were as follows: lncRNA MAGI2‐AS3 (F): 5′‐CACCTTGCTTGACAACTTGA‐3′ and lncRNA MAGI2‐AS3 (R), 5′‐CATTACAGCTCGGCTACTGC‐3′; miR‐374b‐5p (Forward), 5′‐TCAGCGGATATAATACAACCTGC‐3′ and miR‐374b‐5p (Reverse), 5′‐TATCGTTGTTCTCCACTCCTTCAC‐3′; IL‐10 (Forward), 5′‐GAGATCTCCGAGATGCCTTCA‐3′, IL‐10 (Reverse), 5′‐CAAGGACTCCTTTAACAACAAGTTGT‐3′; GAPDH (Forward), 5′‐ACCACAGTCCATGCCATCAC‐3′ and GAPDH (Reverse), 5′‐TCCACCACCCTGTTGCTGTA‐3′; and U6 (Forward), 5′‐ATTGGAACGATACAGAGAAGATT‐3′ and U6 (Reverse), 5′‐GGAACGCTTCACGAATTTG‐3′.

### 2.3 MTT assay for detecting cell viability

Briefly, each cell group was inoculated in 96‐well plates at a density of 2 × 10^5^ cells/well at 37°C and 5% CO_2_. After 48 h, 20 μl of MTT solution (C0009S; Beyotime) was added to each well, followed by incubation for 4 h. After discarding the supernatant, 150 μl of dimethyl sulfoxide was added to the wells, followed by shaking for 10 min. After fully dissolving the crystals, the absorbance value was measured at 570 nm using FLUOstar® Omega Microplate Reader (BMG Labtech GmbH).

### 2.4 Flow cytometry analysis for quantifying cell apoptosis

Briefly, transfected NP cells were cultured into 6‐well plates at a density of 2 × 10^5^ cells/well and incubated at 37°C for 24 h under 5% CO_2_. Then, 5 μl Annexin V (C1062M, Beyotime) was added for 15 min, followed by 5 μL propidium iodide (PI) (C1062M, Beyotime) and mixing. The reaction was performed at room temperature and protected from light for 20 min. Finally, apoptosis was detected on a Cytomics FC‐500 flow cytometer (Beckman Coulter) using Beckman CXP software within 1 h after the reaction.

### 2.5 Caspase3 activity

To determine the activity of Caspase3, Caspase3 Activity Assay Kit (C1116, Beyotime) was used according to the manufacturer's instructions. Briefly, cells were collected by centrifugation at 600*g* at 4°C for 5 min. Then the cells were added with 200 μl lysate, resuspended and precipitated, and lysed in ice bath for 15 min. Subsequently, the supernatant was collected through centrifugation at 16000*g* at 4°C for 10 min, and then the activity of Caspase3 was determined immediately.

### 2.6 ELISA

The supernatant was collected, and inflammatory factors TNF‐α (PT518), IL‐1β (PI305), and IL‐6 (PI330) were examined following instructions of the corresponding ELISA kits (Beyotime). Briefly, the samples were processed, and optical density (OD) values were measured at 450 nm using a spectrophotometer (Thermo Fisher Scientific, Inc.) to determine the content of TNF‐α, IL‐1β, and IL‐6.

### 2.7 Western blot assay

Briefly, cells were lysed with RIPA buffer (P0013C, Beyotime), and total protein was extracted and measured using a BCA protein assay kit. Total protein was separated by 12.5% sodium dodecyl sulfate‐polyacrylamide gel electrophoresis (SDS‐PAGE) and transferred to polyvinylidene fluoride (PVDF) membranes; the membranes were probed with primary antibodies (anti‐GAPDH, 1:1000, #5174, Cell Signaling Technology; anti‐IL‐10, 1:1000, #12163, Cell Signaling Technology; anti‐aggrecan, 1:1000, ab3778, Abcam; anti‐collagen type II, 1:1000, #95855, Cell Signaling Technology) at 4°C overnight. The following day, the PVDF membranes were probed with the secondary antibody (anti‐rabbit IgG, HRP‐linked antibody, 1:1000, #7074, Cell Signaling Technology) at room temperature for 2 h. Subsequently, the West Dura Extended Duration Substrate kit (Thermo Fisher Scientific, Inc.) was used for imaging. Finally, Bandscan 5.0 software was selected to analyze optical density values of electrophoretic bands and perform semi‐quantitative statistical analysis based on grayscale values.

### 2.8 Dual‐luciferase reporter system

Binding sites of lncRNA MAGI2‐AS3 to miR‐374b‐5p and miR‐374b‐5p to IL‐10 were predicted using Starbase and TargetScan software, respectively. The wild‐type sequences (MAGI2‐AS3‐WT, IL‐10‐WT) and mutant‐type sequences (MAGI2‐AS3‐MUT, IL‐10‐MUT) of MAGI2‐AS3/IL‐10 were synthesized by Yeasen and transfected into HEK‐293K cells with mimic control or miR‐374‐5p mimic. After incubation for 48 h at 37°C in 5% CO_2_, luciferase activity was evaluated using a Dual Luciferase Assay Kit (E1910; Promega Corporation) and normalized to *Renilla* luciferase.

### 2.9 Statistical analysis

All experiments were repeated for three times. Data values are presented as mean ± standard deviation from three independent experiments and statistically analyzed using SPSS 21.0 (IBM Corp.). We used the Kolmogorov–Smirnov test to determine the normality of the data in SPSS. Student's *t*‐test was applied to compare two groups, while one‐way analysis of variance was employed to analyze differences between multiple groups. *p* < .05 was considered a statistically significant difference.

## 3 RESULTS

### 3.1 LncRNA MAGI2‐AS3 functioned as a sponge for miR‐374b‐5p

**Figure 1:**
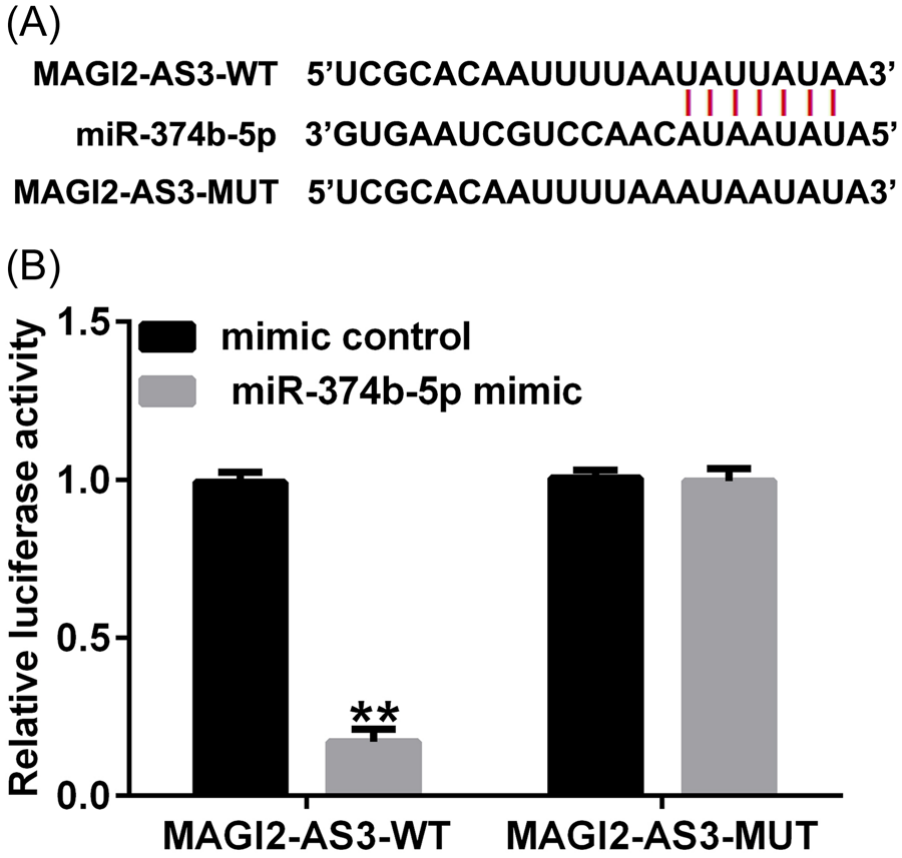
LncRNA MAGI2‐AS3 targets miR‐374b‐5p. (A) Starbase online tool predicted the binding sites between lncRNA MAGI2‐AS3 and miR‐374b‐5p. (B) Dual‐luciferase reporter system revealed the targeted relationship between lncRNA MAGI2‐AS3 and miR‐374b‐5p. *n* = 3. **p < .01 versus Mimic control.

Using Starbase online tool (https://starbase.sysu.edu.cn/), we identified binding sites between lncRNA MAGI2‐AS3 and miR‐374b‐5p (Figure 1A). Subsequently, the targeted relationship between MAGI2‐AS3 and miR‐374b‐5p was verified using the dual‐luciferase reporter assay. As depicted in Figure 1B, compared with the mimic‐control group, cotransfection with miR‐374b‐5p mimic dramatically decreased luciferase activity in the MAGI2‐AS3 WT group. However, no significant differences were noted in the MAGI2‐AS3 MUT groups. These findings suggested that MAGI2‐AS3 was a competing endogenous RNA (ceRNA) for miR‐374b‐5p.

### 3.2 LncRNA MAGI2‐AS3 and miR‐374b‐5p in IDD

**Figure 2:**
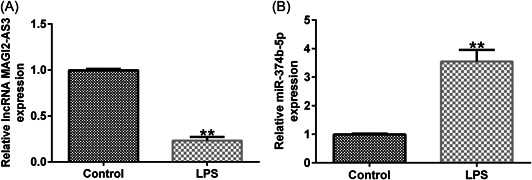
Expression of lncRNA MAGI2‐AS3 and miR‐374b‐5p in the in vitro IDD model. (A) RT‐qPCR assay determined the expression of lncRNA MAGI2‐AS3 in LPS‐induced NP cells. (B) RT‐qPCR assay determined the expression of miR‐374b‐5p in LPS‐induced NP cells. *n* = 3. **p < .01 versus Control. IDD, intervertebral disc degeneration; LPS, lipopolysaccharide; NP, nucleus pulposus; RT‐qPCR, reverse transcription‐quantitative PCR.

To discover the function of MAGI2‐AS3 and miR‐374b‐5p in IDD, we first constructed an in vitro IDD model by treating NP cells with LPS. As shown in Figure 2A, treatment with LPS decreased the MAGI2‐AS3 expression level, whereas miR‐374b‐5p expression was dramatically increased (Figure 2B). Overall, lncRNA MAGI2‐AS3 and miR‐374b‐5p may participate in regulating IDD pathogenesis and development.

### 3.3 LncRNA MAGI2‐AS3 ameliorated injury in IDD by sponging miR‐374b‐5p

**Figure 3:**
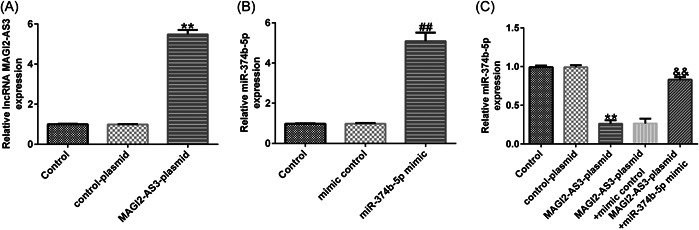
LncRNA MAGI2‐AS3 negatively regulates miR‐374b‐5p expression in NP cells. (A) The RT‐qPCR analysis measured the expression of lncRNA MAGI2‐AS3 after transfection with MAGI2‐AS3‐plasmid. (B) The RT‐qPCR analysis measured the expression of miR‐374b‐5p after transfection with miR‐374b‐5p mimic. (C) The RT‐qPCR analysis measured the expression of miR‐374b‐5p after cotransfection with MAGI2‐AS3‐plasmid and miR‐374b‐5p mimic. *n* = 3. **p < .01 versus Control‐plasmid; ##*p* < .01 versus mimic control; &&*p* < .01 versus MAGI2‐AS3‐plasmid+mimic control. NP, nucleus pulposus; RT‐qPCR, reverse transcription‐quantitative PCR.

Firstly, we confirmed the transfection efficacy of lncRNA MAGI2‐AS3 and miR‐374b‐5p using RT‐qPCR. As presented in Figure 3A, THE MAGI2‐AS3‐plasmid group exhibited increased lncRNA MAGI2‐AS3 expression when compared with that in the control‐plasmid group. Meanwhile, miR‐374b‐5p expression was markedly elevated in the miR‐374b‐5p mimic group when compared with that in the mimic‐control group (Figure 3B). Subsequently, we examined the impact of lncRNA MAGI2‐AS3 on miR‐374b‐5p expression. As depicted in Figure 3C, MAGI2‐AS3‐plasmid transfection dramatically suppressed miR‐374b‐5p expression in NP cells, which was substantially restored following cotransfection with miR‐374b‐5p mimic.

**Figure 4:**
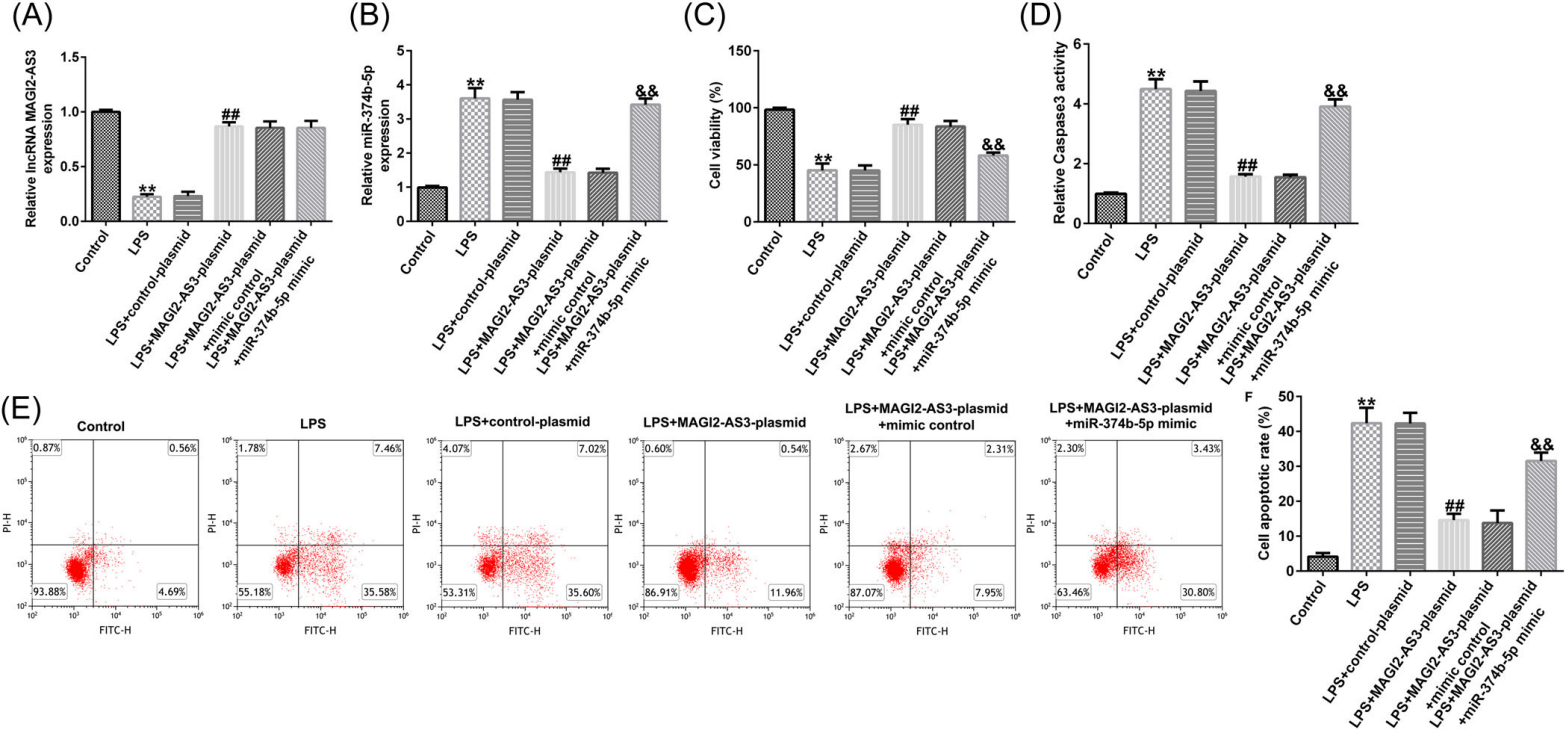
LncRNA MAGI2‐AS3 alleviates injury in LPS‐induced NP cells by suppressing miR‐374b‐5p expression. (A) Expression of lncRNA MAGI2‐AS3 in LPS‐induced NP cells was detected using RT‐qPCR. (B) Expression of miR‐374b‐5p in LPS‐induced NP cells was detected using RT‐qPCR. (C) LncRNA MAGI2‐AS3 rescues the inhibitory effect on NP cell viability induced by LPS treatment by suppressing miR‐374b‐5p expression. (D) LncRNA MAGI2‐AS3 counteracts the promotive effect on Caspase3 activity in NP cells induced by LPS treatment by suppressing miR‐374b‐5p expression. (E and F) LncRNA MAGI2‐AS3 counteracts the promotive effect on NP cell apoptosis induced by LPS treatment by suppressing miR‐374b‐5p expression. *n* = 3. **p < .01 versus Control; ##*p* < .01 versus LPS + control‐plasmid; &&*p* < .01 versus PS + MAGI2‐AS3‐plasmid+mimic control. LPS, lipopolysaccharide; NP, nucleus pulposus; RT‐qPCR, reverse transcription‐quantitative PCR.

Next, NP cells were transfected with control‐plasmid, MAGI2‐AS3‐plasmid, MAGI2‐AS3‐plasmid+mimic control, or MAGI2‐AS3‐plasmid + miR‐374b‐5p mimic. As shown in Figure 4A, LPS‐induced inhibition of MAGI2‐AS3 expression could be partially reversed by MAGI2‐AS3‐plasmid. Moreover, as shown in Figure 4B, WE confirmed that MAGI22‐AS3 could suppress miR‐374b‐5p expression levels in NP cells; this suppression could be markedly counteracted by transfection with miR‐374b‐5p mimic.

The effects of NP cell viability were examined using the MTT assay, flow cytometry, Caspase3 activity, and western blot analysis. As depicted in Figure 4C, treatment with LPS remarkably suppressed NP cell viability. Notably, MAGI2‐AS3 overexpression could reverse the LPS‐induced inhibitory effect on cell viability; however, the effect of MAGI2‐AS3 overexpression on NP cell viability was markedly reversed by miR‐374b‐5p mimic (Figure 4C). Conversely, treatment with LPS notably facilitated NP cell Caspase3 activity and apoptosis Figure 4D−F). Moreover, compared with the LPS group, cell apoptosis was reduced in the LPS + MAGI2‐AS3‐plasmid group; miR‐374b‐5p mimic could counteract the reduced cell apoptosis induced by MAGI2‐AS3 upregulation (Figure 4D−F).

### 3.4 LncRNA MAGI2‐AS3 ameliorated the inflammatory response and ECM degradation in IDD via sponging miR‐374b‐5p

**Figure 5:**
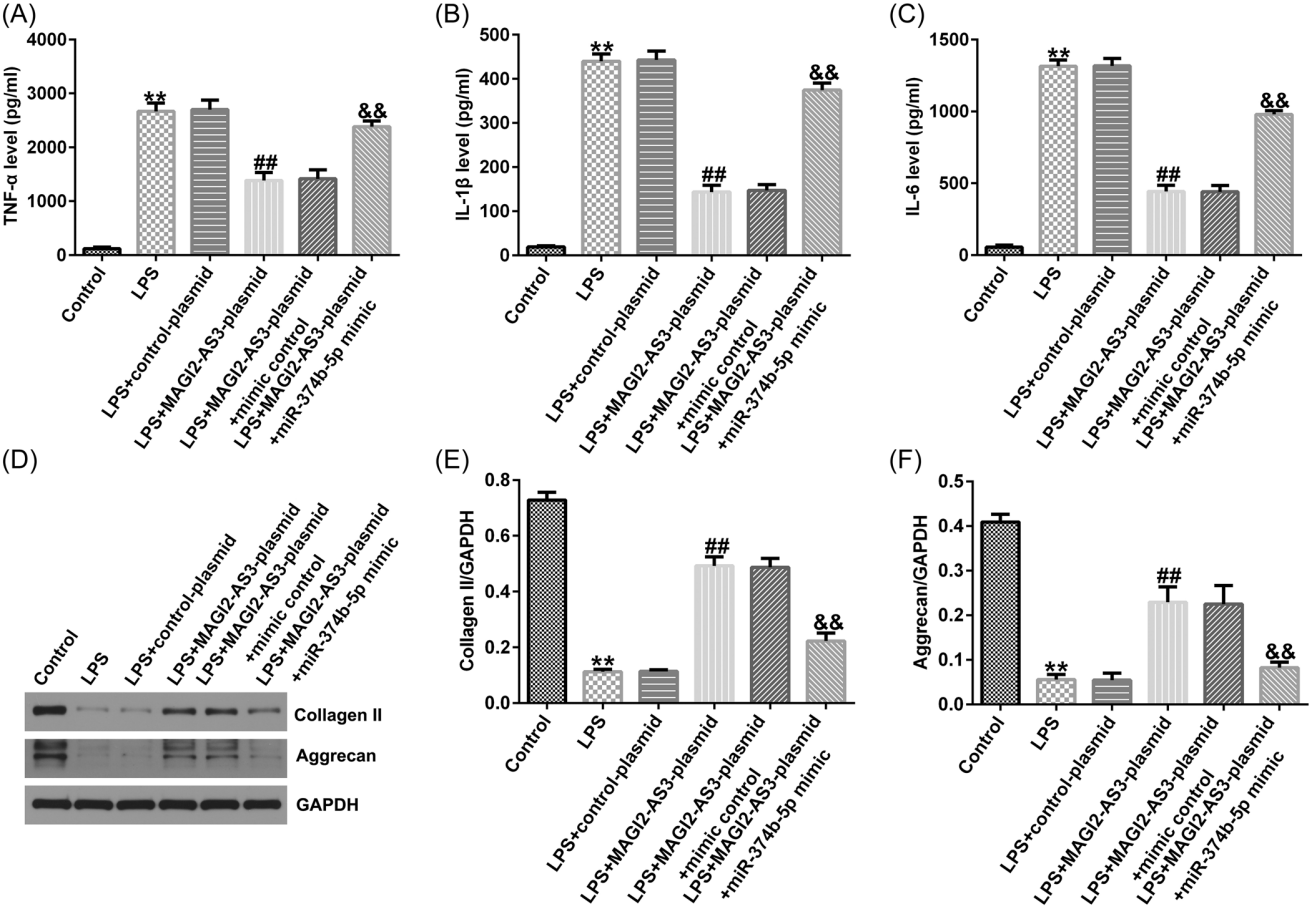
LncRNA MAGI2‐AS3 suppresses the inflammatory response and facilitates extracellular matrix synthesis by inhibiting miR‐374b‐5p expression. (A) ELISA was performed to verify the TNF‐α level. (B) ELISA was performed to verify the IL‐1β level. (C) ELISA was performed to verify the IL‐6 level. (D–F) Protein expression of aggrecan and collagen type II was confirmed by western blot analysis. *n* = 3. **p < .01 *versus* Control; ##*p* < .01 versus LPS + control‐plasmid; &&*p* < .01 versus LPS + MAGI2‐AS3‐plasmid+mimic control. ELISA, enzyme‐linked immunosorbent assay; IL‐1β, interleukin‐1β; Il‐6, interleukin‐6; LPS, lipopolysaccharide; TNF‐α, tumor necrosis factor‐α.

ELISA and western blot analysis were performed to quantify the inflammatory response and ECM degradation in IDD. As illustrated in Figure 5A−C, LPS significantly stimulated TNF‐α, IL‐1β, and IL‐6 secretion in NP cells. Consequently, upregulation of MAGI2‐AS3 could inhibit TNF‐α, IL‐1β, and IL‐6 levels; however, the inhibitory effects were substantially counteracted by miR‐374b‐5p mimic (Figure 5A−C). Likewise, LPS treatment notably reduced ECM‐related markers, collagen type II and aggrecan (Figure 5D–F). Compared with the LPS group, transfection with MAGI2‐AS3‐plasmid enhanced collagen type II and aggrecan expression levels, which was substantially counterbalanced by miR‐374b‐5p upregulation (Figure 5D−F).

### 3.5 IL‐10 was a direct target for miR‐374b‐5p

**Figure 6:**
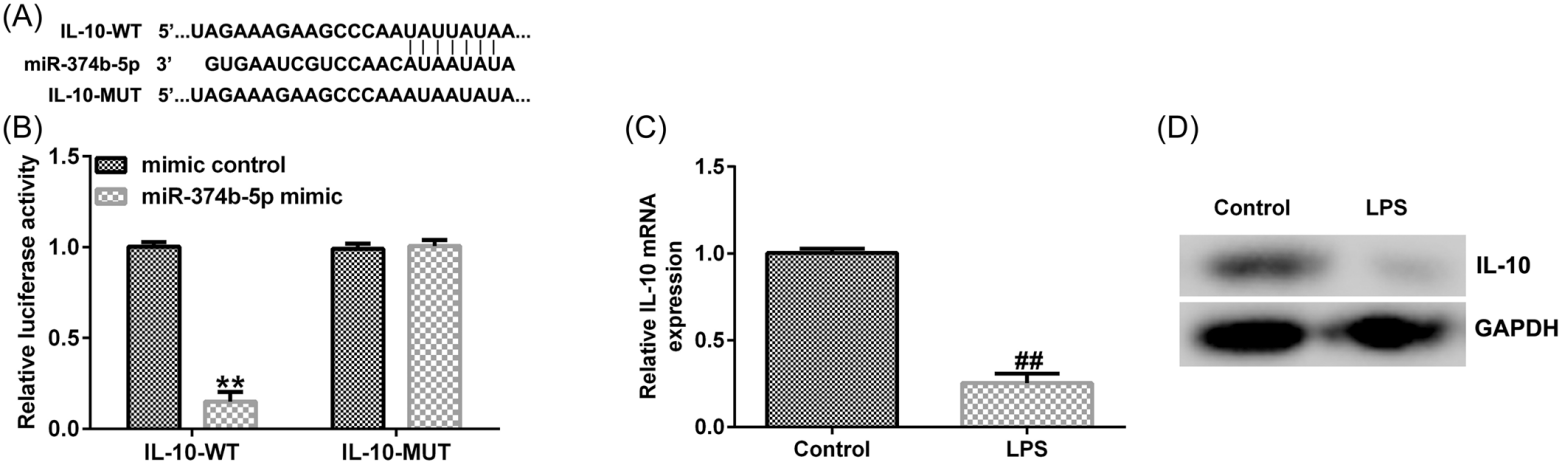
IL‐10 is a downstream target of miR‐374b‐5p. (A) TargetScan online tool displays the binding sequences between IL‐10 and miR‐374b‐5p. (B) Dual‐luciferase reporter assay unveiled the targeted relationship between IL‐10 and miR‐374b‐5p. (C) RT‐qPCR experiment measured the mRNA level of IL‐10 in LPS‐treated NP cells. (D) Western blot analysis measured protein levels of IL‐10 in LPS‐treated NP cells. *n* = 3. **p < .01 versus Mimic control; ##*p* < .01 versus Control. IL‐10, interleukin‐10; LPS, lipopolysaccharide; NP, nucleus pulposus; RT‐qPCR, reverse transcription‐quantitative PCR.

**Figure 7:**
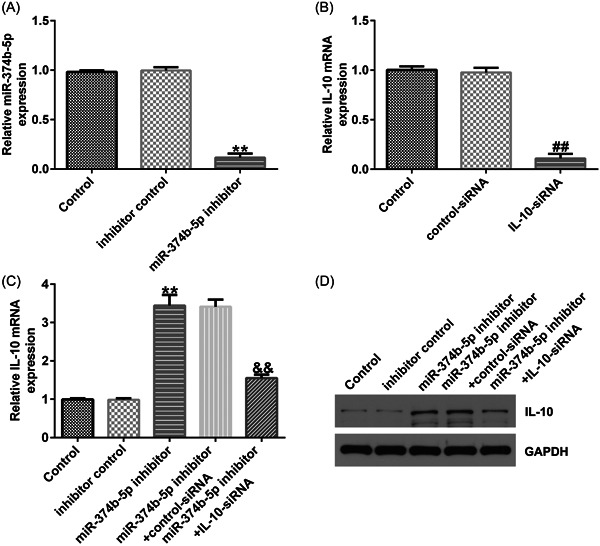
miR‐374b‐5p knockdown facilitates IL‐10 expression in NP cells. (A) Transfection efficacy of miR‐374b‐5p inhibitor was examined using RT‐qPCR analysis. (B) Transfection efficacy of IL‐10‐siRNA was examined using RT‐qPCR analysis. (C) Expression of IL‐10 in NP cells after cotransfection with miR‐374b‐5p inhibitor and IL‐10‐siRNA was examined using RT‐qPCR analysis. *n* = 3. **p < .01 versus Inhibitor control; ##*p* < .01 versus Control‐siRNA; &&*p* < .01 versus miR‐374b‐5p inhibitor+control‐siRNA. IL‐10, interleukin‐10; LPS, lipopolysaccharide; NP, nucleus pulposus; RT‐qPCR, reverse transcription‐quantitative PCR.

We conducted a series of experiments to verify the targeted association between IL‐10 and miR‐374b‐5p. First, binding sequences between miR‐374b‐5p and IL‐10 were predicted using TargetScan (Figure 6A). Subsequently, using the dual‐luciferase reporter assay, we revealed that luciferase activity was markedly reduced in the miR‐374b‐5p mimic and IL‐10 WT cotransfection groups (Figure 6B). In contrast, no significant differences were detected in IL‐10 MUT‐transfection groups. The IDD in vitro model exhibited a prominent decrease in IL‐10 mRNA and protein expression levels (Figure 6C−D). Then, IL‐10‐siRNA and miR‐374b‐5p inhibitors were transfected in LPS‐treated NP cells. As depicted in Figure 7A, miR‐374b‐5p expression was dramatically reduced in the miR‐374b‐5p inhibitor group when compared with that in the inhibitor‐control group. Meanwhile, IL‐10 expression was inhibited in the IL‐10‐siRNA group when compared with that in the control‐siRNA group (Figure 7B). Moreover, miR‐374b‐5p inhibitor‐transfection elevated IL‐10 expression; however, this elevation was substantially reversed by cotransfection with IL‐10‐siRNA (Figure 7C).

### 3.6 miR‐374b‐5p silencing alleviated IDD development by targeting IL‐10

**Figure 8:**
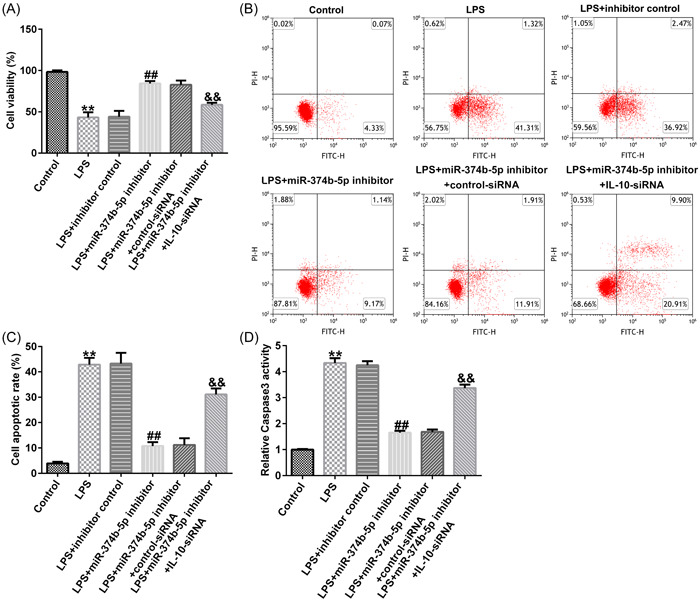
Silencing of miR‐374b‐5p ameliorates LPS‐induced cell injury in NP cells by targeting IL‐10. (A) Silencing of miR‐374b‐5p facilitates NP cell viability under LPS treatment by targeting IL‐10. (B−C) Silencing of miR‐374b‐5p suppresses NP cell apoptosis under LPS treatment by targeting IL‐10. (D) Silencing of miR‐374b‐5p suppresses Caspase3 activity under LPS treatment by targeting IL‐10. *n* = 3. **p < .01 versus Control; ##*p* < .01 versus LPS + inhibitor control; &&*p* < .01 versus LPS + miR‐374b‐5p inhibitor + control‐siRNA. IL‐10, interleukin‐10; LPS, lipopolysaccharide.

**Figure 9:**
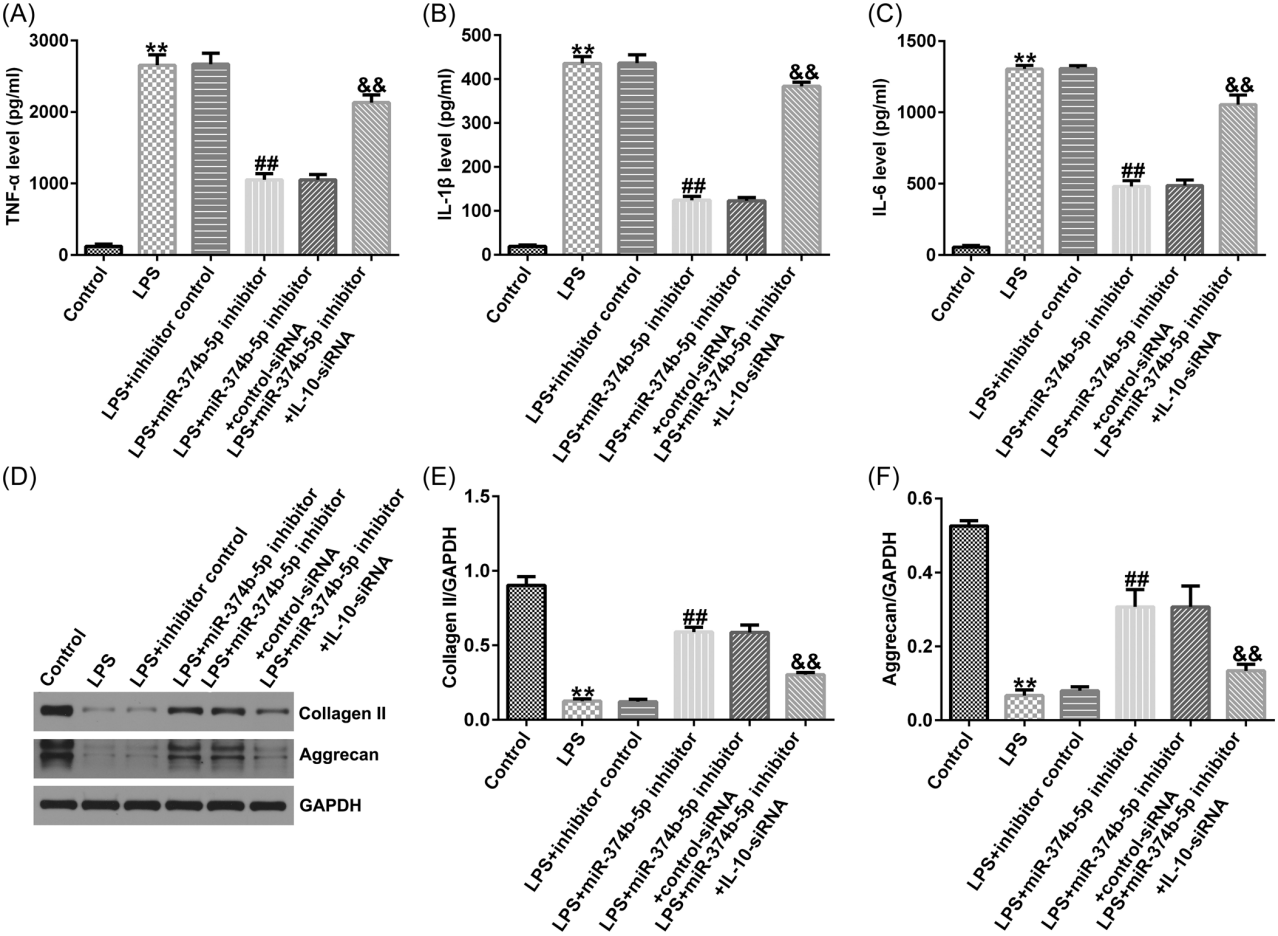
Downregulation of miR‐374b‐5p suppresses the inflammatory response and facilitates extracellular matrix synthesis in IDD by targeting IL‐10. (A) TNF‐α level detected using ELISA. (B) IL‐1β expression level detected using ELISA. (C) IL‐6 expression level detected using ELISA. (D−F) Protein expression of ECM‐related biomarkers aggrecan and collagen type II was quantified by RT‐qPCR analysis. *n* = 3. **p < .01 versus Control; ##*p* < .01 versus LPS + inhibitor control; &&*p* < .01 versus LPS + miR‐374b‐5p inhibitor+control‐siRNA. ECM, extracellular matrix; ELISA, enzyme‐linked immunosorbent assay; IDD, intervertebral disc degeneration; IL‐1β, interleukin‐1β; IL‐6, interleukin‐6; IL‐10, interleukin‐10; LPS, lipopolysaccharide; RT‐qPCR, reverse transcription‐quantitative PCR; TNF‐α, tumor necrosis factor‐α.

To verify the role of the miR‐374b‐5p/IL‐10 axis in IDD development, we transfected inhibitor control, miR‐374b‐5p inhibitor, control‐siRNA, and IL‐10‐siRNA in LPS‐treated NP cells. As depicted in Figure 8A−D, THE LPS‐induced decreased cell viability, increased cell apoptosis, and Caspase3 activity in NP cells could be substantially rescued by miR‐374b‐5p inhibitor. Similarly, LPS‐induced elevated expression levels of TNF‐α, IL‐1β, and IL‐6 were notably reduced by silencing miR‐374b‐5p expression (Figure 9A−C). Based on the western blot analysis, LPS‐induced reductions in collagen type II and aggrecan expression could be enhanced by transfection with miR‐374b‐5p inhibitor (Figure 9D−F). IL‐10‐siRNA could reverse all the above results triggered by the miR‐374b‐5p inhibitor (Figures 8 and 9). Taken together, miR‐374b‐5p downregulation could mitigate LPS‐induced NP cell injury, inflammatory response, and ECM degradation by targeting IL‐10.

## 4 DISCUSSION

Healthy intervertebral disc cells contain a small number of cells and are sparsely distributed.^27^ An important feature of IDD is the presence of NP cell clusters, and the more severe the damage, the more pronounced the abnormal cell growth. It has been suggested that excessive apoptosis and suppressed proliferation of NP cells are important underlying factors for IDD development.^1^ In addition, IDD leads to the release of inflammatory factors, and thus leading to abnormal proliferation and apoptosis of intervertebral disc cells, resulting in an imbalance in the synthesis and breakdown of ECM, which, in turn, causes IDD.^7^, ^28^ In the present study, we treated NP cells with LPS to construct an in vitro IDD model. Following exposure to LPS, we detected the presence of reduced NP cell proliferation and ECM synthesis, along with elevated cell apoptosis and proinflammatory cytokines production, consistent with the known characteristics of IDD.

LncRNA MAGI2‐AS3 is a recently identified lncRNA, which has been widely examined as a tumor suppressor in various cancers, including hepatocellular carcinoma, bladder, lung, ovarian, cervical, and prostate cancers.^29^, ^30^, ^31^, ^32^, ^33^, ^34^ LncRNA MAGI2‐AS3 plasma levels have been evaluated in patients with IDD pre‐ and post‐treatment, and the findings indicated that patients with IDD were found to exhibit reduced lncRNA MAGI2‐AS3 expression, and its expression was significantly augmented after clinical treatments.^15^ Herein, our results confirmed that lncRNA MAGI2‐AS3 expression was reduced in LPS‐triggered NP cells, and augmenting this expression could promote NP cell viability and ECM synthesis while suppressing apoptosis and inflammation. Hence, the findings of our study suggest the protective impact of lncRNA MAGI2‐AS3 in IDD pathogenesis.

LncRNAs can function its roles as endogenous miRNA sponges in diseases.^16^ In this study, using the Starbase online tool, miR‐374b‐5p was predicted as a target for lncRNA MAGI2‐AS3. Subsequently, our study validated the targeted association between miR‐374b‐5p and lncRNA MAGI2‐AS3 via functional experiments. miR‐374b‐5p has been widely investigated in various cancers, cardiovascular diseases, and immune‐related diseases.^35^, ^36^, ^37^, ^38^, ^39^, ^40^ Moreover, Akbaba et al.^41^ have elaborated that miR‐374b‐5p acts as an inflammation‐related gene, indicating that miR‐374b‐5p may participate in inflammation‐related diseases. Furthermore, miR‐374b‐5p was shown to be elevated in postmenopausal osteoporosis, and its upregulation could suppress osteoblast differentiation and bone formation.^22^ Herein, miR‐374b‐5p expression was dramatically increased in the IDD in vitro model. Moreover, silencing miR‐374b‐5p could facilitate NP cell growth and ECM synthesis while suppressing the inflammatory response. To the best of our knowledge, our study is the first to illustrate that miR‐374b‐5p knockdown could relieve IDD development, functioning as a feasible therapeutic biomarker for IDD.

miRNA functions its roles through preventing mRNA translation by binding to the 3‐terminal noncoding region of its target mRNAs. To exploare the mechanism of the role of miR‐374b‐5p in IDD, we determined the potential targtes of miR‐374b‐5p. Using TargetScan, dual‐luciferase reporter assay, and rescue experiments, we confirmed that IL‐10 was a potential target for miR‐374b‐5p. As a common anti‐inflammatory cytokine, it has been extensively reported that IL‐10 can ameliorate immune response.^42^, ^43^, ^44^ Moreover, the anti‐IDD role of IL‐10 has received considerable attention. For instance, IL‐10 overexpression could provide protection against IDD development.^45^, ^46^ Ge et al.^47^ have reported that IL‐10 could ameliorate NP degradation by inducing collagen II and aggrecan levels. Consistent with previous reports,^45^, ^46^, ^47^ our study noted reduced IL‐10 expression in LPS‐induced NP cells. Moreover, IL‐10 silencing could blunt the protective role of miR‐374b‐5p on IDD.

Taken together, the current study proved for the first time that lncRNA MAGI2‐AS3 can play a protective role in LPS induced IDD in vitro model by regulating the miR‐374b‐5p/IL‐10 signal axis. This study provides a potential target for the clinical diagnosis and treatment of IDD, and provides more theoretical basis for the study of the pathogenesis of IDD. However, there were also some limitations of this study. For example, because of the complexity of the pathogenesis of IDD, the inclusion of primary NP cells from IDD patients into the study will make our results more convincing. Besides, this study did not investigate the effect of lncRNA MAGI2‐AS3/miR‐374b‐5p on IDD animal models. Moreover, the sample size of this study is small.

## 5 CONCLUSIONS

LncRNA MAGI2‐AS3 can act as ceRNA to upregulate IL‐10 expression by sponging miR‐374b‐5p, thereby alleviating cell injury, inflammation, and ECM degradation in IDD. Therefore, lncRNA MAGI2‐AS3 can be a novel target for IDD detection and provide a scientific basis for IDD therapeutic strategy.

## AUTHOR CONTRIBUTIONS

Jiang Yu contributed to the study design, data collection, statistical analysis, data interpretation and manuscript preparation. Chengjin Li contributed to data collection and statistical analysis. All authors read and approved the final manuscript.

## CONFLICT OF INTEREST STATEMENT

The authors declare no conflict of interest.

## Data Availability

The datasets used and/or analyzed during the current study are available from the corresponding author on reasonable request.
